# Angiotensin Converting Enzyme Inhibitory and Antioxidant Activities of Enzymatic Hydrolysates of Korean Native Cattle (Hanwoo) Myofibrillar Protein

**DOI:** 10.1155/2017/5274637

**Published:** 2017-12-17

**Authors:** Seung Yun Lee, Sun Jin Hur

**Affiliations:** Department of Animal Science and Technology, Chung-Ang University, 4726 Seodong-daero, Daedeok-myeon, Anseong-si, Gyeonggi-do 17546, Republic of Korea

## Abstract

The purpose of this study was to determine the angiotensin converting enzyme (ACE) inhibitory and antioxidant activities of myofibrillar protein hydrolysates (HMPHs) of different molecular weights (<3 and <10 kDa) derived from Korean native cattle (Hanwoo breed) using a commercially available and inexpensive enzyme (Alkaline-AK). HMPH of both tested molecular weights had ACE inhibitory activity. Among the antioxidant activities, iron chelation and nitrite scavenging activities were higher in low-molecular-weight peptide of HMPH (<3 kDa), whereas 2,2-diphenyl-1-picrylhydrazyl (DPPH) radical scavenging activity was higher in high-molecular-weight peptide of HMPH (<10 kDa). HMPH did not induce cytotoxicity in RAW 264.7 cells at concentrations of 5–20 mg/mL. These results indicate that HMPH can be cheaply produced using Alkaline-AK and applied as a potential ACE inhibitor and antioxidant.

## 1. Introduction

Numerous studies have focused on bioactive peptides isolated from food sources and their diverse properties such as antioxidant, hypotensive, antimicrobial, immunomodulatory, antithrombotic, and opioid receptor-activating activities [1–3]. Bioactive peptides have been produced from food sources using various proteases such as pepsin, trypsin alcalase, neutrase, and thermolysin [1, 4, 5]. Angiotensin converting enzyme (ACE) inhibitory and antioxidative peptides can be obtained from meat protein, which contains a high abundance of certain essential amino acids that are sparse in plant protein [6, 7]. Many ACE inhibitors have been isolated by the enzymatic digestion of meat protein from sources such as chicken muscle [4, 8]. Several studies [6, 9, 10] have reported that antioxidant peptides derived from meat protein are safer than synthetic antioxidant agents for* in vivo *application. However, few studies on peptides derived from beef protein have been reported.

Hanwoo* (Bos taurus coreanae)* is a cattle breed native to Korea that settled in the Korean Peninsula around 4000 BC and originated as a hybrid of* Bos taurus* ×* Bos zebu* [11]. Hanwoo can be used as an adequate protein source because its meat contains all the essential amino acids needed by humans [12, 13]. Bioactive peptides have been produced from meat protein via enzymatic hydrolysis using pepsin, trypsin, alcalase, neutrase, or thermolysin. However, these enzymes have a high cost and a low hydrolytic efficiency. Therefore, the purpose of this study was to determine the chemical and free amino acid compositions of myofibrillar protein hydrolysates of different molecular weights derived from Hanwoo (HMPH) by using a cost-effective industrial enzyme and to determine the ACE inhibitory and antioxidant activities of HMPH.

## 2. Materials and Methods

### 2.1. Materials

Tris/sodium dodecyl sulfate/glycine (SDS) buffer, Laemmli buffer, Bio-Safe™ Coomassie G250 stain, Mini-PROTEAN® precast gels, and Precision Plus Protein™ Standards were employed for the separation of proteins by SDS-polyacrylamide gel electrophoresis (PAGE). ACE, hippuryl-l-histidyl-l-leucine, trifluoroacetic acid, ferrozine, DPPH, disodium ethylenediaminetetraacetate, and 2,2′-azino-*bis*(3-ethylbenzothiazoline-6-sulfonic acid) diammonium salt (ABTS) and 3-(4,5-dimethylthiazol-2-yl)-2,5-diphenyltetrazolium bromide (MTT) were purchased from Sigma-Aldrich (St. Louis, MO, USA). Roswell Park Memorial Institute (RPMI) 1640 medium was obtained from Welgene Inc. (Daegu, Korea). Penicillin, streptomycin, and fetal bovine serum (FBS) were purchased from Life Technologies (Carlsbad, CA, USA).

### 2.2. Proximate Analysis of Myofibrillar Protein and HMPH

Moisture content was assessed by oven dry for 20 h at 110°C. Crude protein content was determined by AOAC method (2000). Crude lipid content was measured by the ether extraction method. Crude ash content was assessed by heating samples in a muffler furnace at 550°C for 4 h (AOAC, 2000). Amino acids were analyzed using an amino acid analyzer (Model 835–50, Hitachi, Tokyo, Japan) with a C18 column (4.6 × 250 mm, 5 *μ*m, Waters, Milford, MA, USA). The resulting samples was determined at 38°C and detected a flow rate of 1.0 mL/min at 254 nm. All amino acids analyses were determined in triplicate.

### 2.3. Preparation of Myofibrillar Protein and HMPH

The 500 g of Hanwoo beef shank (pH 5.8) (with whole muscle including* Ulnaris lateralis, Triceps surae, Superficialis digital flexor,* and* Brachialis*) which was purchased from local market (Anseong-si, Gyeonggi, Korea) was added to 5 L distilled water (DW) and washed 10 times to remove blood and fat. Sample was extracted with 4 L of 0.04 M phosphate buffer (pH 7.4). Meat and buffer were homogenized using a homogenizer for 1 min, and water was eliminated to obtain myofibrillar protein by centrifugation (3,000 ×g, 15 min). The extraction procedure was repeated seven times. The protease Alkaline-AK (180–200 KU/g solid) was obtained by fermenting soybean meal with* Bacillus methylotrophicus*, and the obtained myofibrillar protein was hydrolyzed with 0.2% Alkaline-AK (w/w) at 60°C and pH 11 for 5 h. Then, the hydrolysate was performed to inactivate the Alkaline-AK at 100°C for 15 min. Hydrolysates were processed using ultrafiltration devices with <3 kDa and <10 kDa upper mass cut-off membranes (Amicon® Ultra, Millipore, Billerica, MA, USA) to produce low-molecular-weight peptide of HMPH (HMPH3) and high-molecular-weight peptide of HMPH (HMPH10), respectively.

### 2.4. Protein Concentration and SDS-PAGE

SDS-PAGE was performed according to the methods of Meyer and Lamberts (1965) [14]. The protein content of HMPH was measured with a protein assay reagent. The protein standard was used for bovine serum albumin (BSA) and the molecular weight changes of the HMPH were determined by SDS-PAGE on a 12% polyacrylamide gel at a constant voltage of 80 V for 2 h. The resulting samples were stained with Coomassie Brilliant Blue R-250 (Bio-Rad Laboratories).

### 2.5. Measurement of ACE Inhibitory (ACEI) Activity of HMPH

The ACEI activity of peptides of HMPH was measured by modified Cushman and Cheung (1971) methods [15], with slight modifications. Fifty microliters of peptides of HMPH solution with 50 *μ*L of substrate (0.004 M hippuryl-l-histidyl-l-leucine in 0.05 M sodium borate buffer, pH 8.3) was preincubated (15 min at 37°C) and then incubated with 100 *μ*L of ACE solution (0.025 U/mL) for 1 h at 37°C. The control and blank contained DW instead of sample. The reaction was completed with the addition of 250 *μ*L of 1 M HCl. The hippuric acid of resulting samples was extracted with 500 *μ*L of ethyl acetate. After centrifugation (5,000 rpm, 10 min), 0.25 mL of the supernatant was dried at 80°C for 1 h. The extracted hippuric acid was dissolved in 1 mL of DW and the absorbance was detected at 228 nm using a spectrophotometer (Jasco, Tokyo, Japan). The ACEI activity was calculated as follows: ACEI activity (%) = (*C* − *S*)/(*C* − *B*) × 100, where *C* is the control, *S* is the sample, and *B* is the blank.

### 2.6. Measurement of Antioxidant Activities of HMPH

#### 2.6.1. ABTS Radical Scavenging Assay

The ABTS free radical scavenging activities of peptides of HMPH3 and HMPH10 at 5, 10, 20, or 40 mg/mL were determined using a previously described method [16]. The ABTS^•^^+^ radical was produced by mixing 7 mM ABTS with 2.45 mM potassium persulfate. The reaction mixture was left to stand for 12 h in the dark. The ABTS^•^^+^ solution was diluted with PBS (pH 7.4) to adjust the absorbance at 734 nm to 0.70 ± 0.02. The 10 *μ*L of peptides of HMPH3 or HMPH10 at 5, 10, 20, or 40 mg/mL was mixed with 990 *μ*L of ABTS^•^^+^ solution and mixed for 5 min. The ABTS^•^^+^ scavenging activity was determined using by spectrophotometer at 734 nm. The controls used extraction solutions instead of HMPH. The radical scavenging activity was calculated as

(1) ABTS•+ scavenging  activity%=1−samplecontrol×100.

#### 2.6.2. DPPH Radical Scavenging Activity

The DPPH radical scavenging activity of peptides of HMPH was measured using colorimetric method [17]. Fifty *μ*L of peptides of HMPH3 or HMPH10 at 5, 10, 20, or 40 mg/mL was mixed with 50 *μ*L of DPPH reagent in methanol. The reaction mixture incubated in the dark for 25 min. The DPPH scavenging activity was measured at 517 nm using a Sunrise™ microplate reader. Controls were prepared in the same manner using DW instead of sample. The DPPH radical scavenging activity was determined as

(2) DPPH• scavenging  activity%=1−samplecontrol×100.

#### 2.6.3. Iron Chelating Activity

The iron chelating ability of peptides of HMPH was assessed using the modified previously method [18]. In the chelation test, 500 *μ*L of peptides of HMPH3 or HMPH10 at 5, 10, 20, or 40 mg/mL was mixed with 100 *μ*L of FeCl_2_ (600 *μ*M) and 0.9 mL of methanol was added to the mixed sample and then mixed for 1 min. The mixture was reacted for 10 min. Subsequently, 0.1 mL of 5 mM ferrozine solution was added to the mixed sample, and the mixture was reacted for 10 min. The resulting sample was assessed at 562 nm using a Sunrise microplate absorbance reader (Tecan). The control sample contained 500 *μ*L DW, 100 *μ*L FeCl_2_, and 100 *μ*L of 5 mM ferrozine solution. The iron chelating activity was calculated as

(3) $$\begin{array}{c} \text{Chelating activity}\left(\;\%\;\right)=\left[\;1-\frac{\left(sample\right)}{\left(control\right)}\;\right]\times 100. \end{array}$$

#### 2.6.4. Nitrite Scavenging Activity

The nitrite scavenging activity of peptides of HMPH was determined using a previous method [19]. One milliliter of peptides of HMPH3 or HMPH10 solution (5, 10, 20, or 40 mg/mL) was mixed with 0.5 mL of 2 mM sodium nitrite (NaNO_2_) with 100 mM HCl buffer (adjusted to pH 2.0) and then topped up to 5 mL. After incubation at 37°C for 1 h, 50 *μ*L of this solution was mixed with 250 *μ*L DW and 50 *μ*L Griess reagent and kept for 20 min. The absorbance was assessed at 540 nm. The nitrite scavenging activity was calculated as

(4) Nitrite  scavenging  activity%=1−samplecontrol×100.

### 2.7. Effect of HMPH on Cell Viability

#### 2.7.1. Cell Culture

RAW 264.7 murine macrophages cells were purchased from ATCC (Manassas, VA, USA). The RAW 264.7 cells were cultured with RPMI 1640 medium added to 1% penicillin (100 IU/mL), streptomycin, and 10% FBS, in a humidified atmosphere containing 5% CO_2_ at 37°C.

#### 2.7.2. Cell Viability Analysis

The effect of peptides of HMPH on cell viability was assessed by MTT assay. Briefly described, RAW 264.7 cells (5 × 10^4^ cells/well) were placed in 96-well culture plates and then incubated at 37°C under 5% CO_2_. After 24 h incubation, cells were treated with peptides of HMPH3 or HMPH10. Next, 0.5 mg/mL of MTT reagent was added to 96-well plate well and the samples were then incubated for 4 h at 37°C. After a wash step, the formazan dye precipitates were dissolved in 0.1 mL dimethyl sulfoxide (DMSO). The resulting samples were measured at 570 nm using a microplate reader.

#### 2.7.3. Cell Morphology Analysis

RAW 264.7 cells (1 × 10^6^ cells/mL) were plated in 6-well plates. After 24 h incubation, the cells were treated with peptides of HMPH3 or HMPH10 at different concentrations. After 24 h, the cells were observed under bright field microscopy.

### 2.8. Statistical Analysis

All statistical analyses were determined using the one-way analysis of variance procedure of SPSS 20.0 (IBM, Armonk, NY, USA). A Student Newman Keuls (SNK) multiple comparisons test was used to evaluate significant differences between mean values, and data were based on a significance level of *p* < 0.05.

## 3. Results

**Figure 1:**
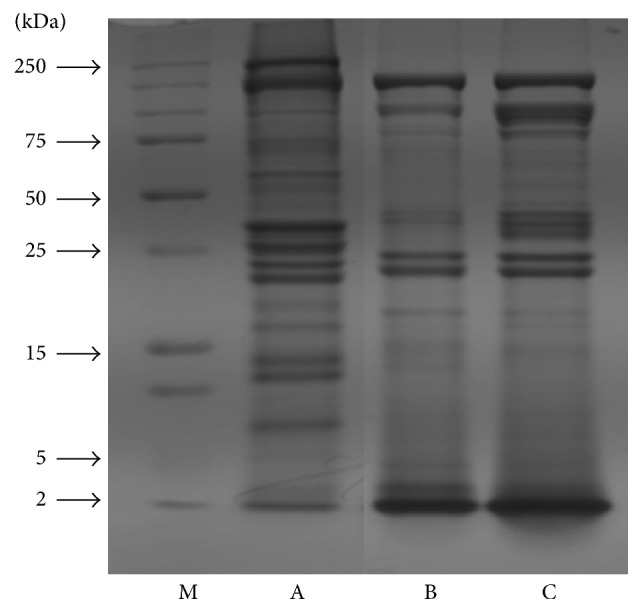
Changes in SDS-PAGE of HMPHs (M: marker, A: myofibrillar protein without enzyme hydrolysis, B: hydrolysate with pepsin, and C: hydrolysate with Alkaline-AK).

The chemical compositions of myofibrillar protein and HMPH are shown in Table 1. The crude protein contents of myofibrillar protein and HMPH were 26.99% and 73.62% of wet and dry matter, respectively. Therefore, the recovery rate of HMPH was approximately 5.5% of wet matter. The amino acid compositions of myofibrillar protein and HMPH were determined using amino acid analyzer (data not shown). Aspartic acid (ASP), glutamic acid (Glu), lysine (Lys), and leucine (Leu) were the most abundant amino acids in myofibrillar protein and peptides of HMPH (both 3 and 10 kDa). The molecular weight change of proteins in HMPH was evaluated by SDS-PAGE. The hydrolysis patterns of pepsin (used as a control) and Alkaline-AK were similar. Pepsin and Alkaline-AK almost completely hydrolyzed the proteins, and a low-molecular-weight band was observed at the bottom of the gel (<2 kDa). These results indicate that the hydrolysis efficiency of Alkaline-AK seems to be the same as that of pepsin (Figure 1).

**Figure 2:**
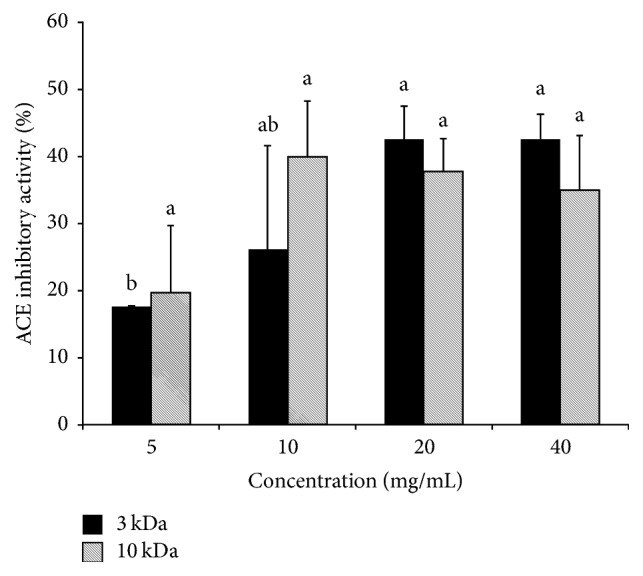
ACE inhibitory activity of HMPH. No significant differences were detected between HMPH samples of different molecular weights (<3 kDa versus <10 kDa). ^a-b^Means with different letters differed significantly according to the concentration of HMPH (*p* < 0.05).

The HMPH3 and HMPH10 had ACEI activities in the ranges of 17.63–42.56% and 19.69–39.95%, respectively (Figure 2). The ACEI activity between HMPH3 and HMPH10 did not show significant difference.

**Figure 3:**
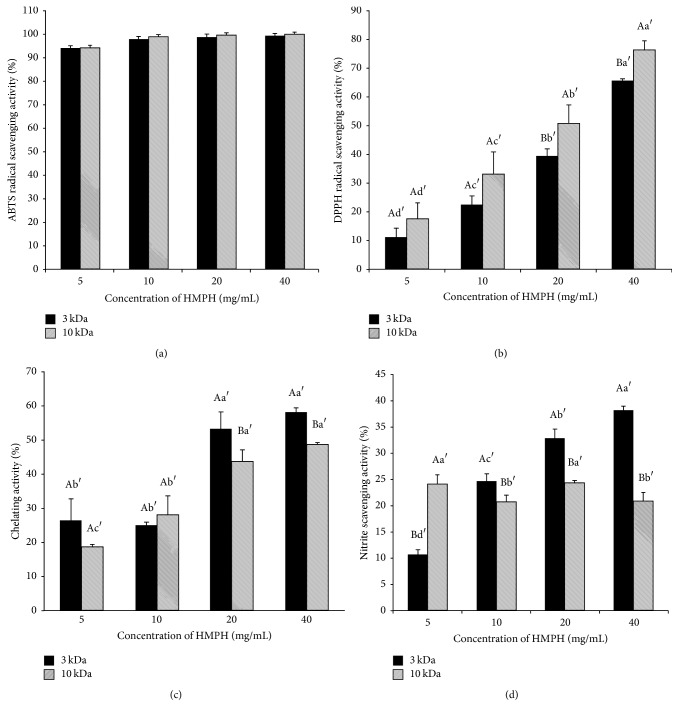
Antioxidant activities of HMPH. (a) ABTS radical scavenging activity, (b) DPPH radical scavenging activity, (c) iron chelating activity, and (d) nitrite scavenging activity. Data are given as mean values ± standard deviation (*n* = 3). ^A–B^Means with different letters differed significantly according to the molecular weight of HMPH (<3 kDa versus <10 kDa) (*p* < 0.05). ^a′–d′^Means with different letters differed significantly according to the concentration of HMPH (*p* < 0.05).

The ABTS radical scavenging activity was dose-dependent and was more than 90% at all tested doses of HMPH (Figure 3(a)). The ACEI activity between HMPH3 and HMPH10 did not show significant difference.

HMPH showed DPPH radical scavenging activity, which varied depending on the molecular weight and dosage of HMPH (Figure 3(b)). DPPH radical scavenging activity was higher in HMPH10 than in HMPH3.

The ion chelating and nitrite scavenging activities of HMPH are shown in Figures 3(c) and 3(d). HMPH3 had significantly higher iron chelating and nitrite scavenging activities than HMPH10. Moreover, the iron chelating and nitrite scavenging activities of HMPH increased with concentration.

**Figure 4:**
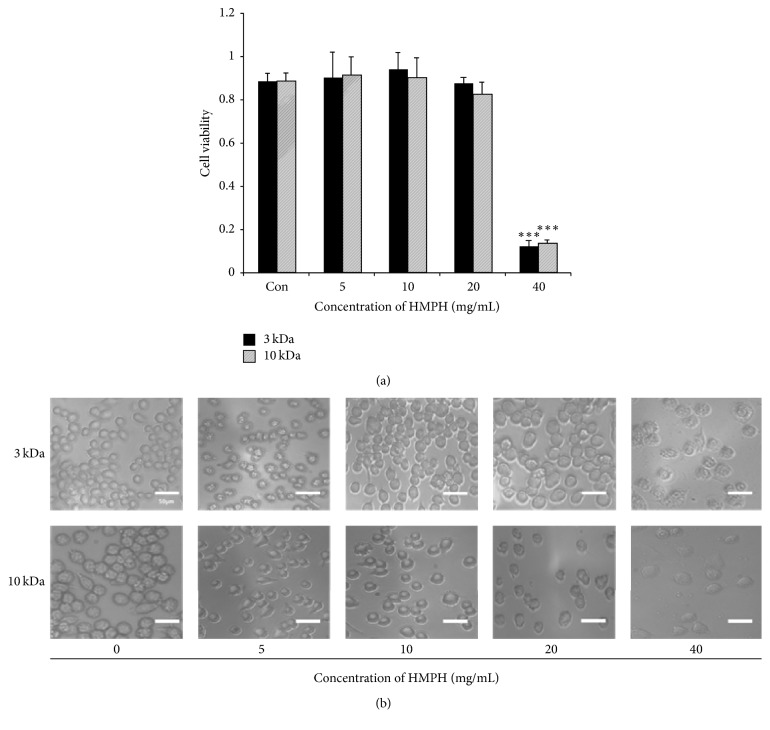
Effect of HMPH on the viability of RAW 264.7 cells. Data are given as mean values ± standard deviation (*n* = 3). ^*∗∗∗*^*p* < 0.001 compared with control cells. (b) Cell morphology was assessed by light microcopy (×200 magnification) after cells were treated with HMPH (0–40 mg/mL). Scale bar = 50 *μ*m.

The cell viability of the RAW 264.7 macrophage cell line was determined using MTT assay (Figure 4). Neither HMPH3 nor HMPH10 affected the viability of RAW 264.7 cells at doses of 5–20 mg/mL, whereas cell viability decreased at HMPH doses above 40 mg/mL.

## 4. Discussion

This study determined the amino acid compositions of myofibrillar protein and HMPH of different molecular weights. The main amino acids in HMPH were Asp, Glu and Lys (a hydrophilic amino acid), Leu, and alanine (a hydrophobic amino acid). The amino acid composition was not different between myofibrillar protein and HMPH in this study. A previous study [20] studied that a novel ACEI peptide from porcine skeletal muscle troponin was comprised of Glu, Lys, and arginine (Arg). Leu, which is a hydrophobic amino acid, has also been reported as a constituent of ACEI and antioxidative peptides [2, 20]. In our previous study [21] we found that diverse peptides have ACEI activity, which is highly related to the degree of hydrolysis and the peptide sequence. ACEI peptides often had hydrophobic amino acids at the last position of the N-terminus and proline residues in peptides derived from animal products. The hydrolysis degree and peptide sequence depend on the peptide source, enzyme type, and hydrolysis conditions; thus, ACE inhibition is also influenced by these variables.

This study revealed that the ACEI activity of peptides of HMPH depends on amino acids. In this study, HMPH3 showed a higher ACEI activity than HMPH10 at above 20 mg/mL. Although the exact mechanisms contributing to the ACEI activity of peptides of HMPH with different molecular weights are currently unknown, several possible mechanisms could be involved. Yamada et al. (2002) showed that the antihypertensive activity of ACE inhibitors comprised of long-chain peptides can result from them being processed through hydrolysis into shorter, active fragments. Shorter peptide particle can be absorbed from the intestine and then immediately interact with the suitable receptors [22]. Therefore, we assume that HMPH3 could more easily combine with a substrate (e.g., hippuryl-l-histidyl-l-leucine or* o*-phthaldialdehyde) than HMPH10 could. This could explain why HMPH3 had a stronger inhibitory effect on ACE activity than HMPH10 did in this study. Ryan et al. and Zeng et al. [2, 23] reported on prodrug type inhibitors that are released by ACE or proteases. For instance, the peptide LKPNM (Leu-Lys-Pro-Asn-Met) had a 50% inhibitory concentration (IC_50_) of 2.40 mol·dm^−3^. LKPNM was hydrolyzed to produce a peptide (Leu-Lys-Pro) with an ACEI activity approximately 8 times higher than the precursor peptide (IC_50_ = 0.32 mol·dm^−3^) [24]. This result indicates that low-molecular-weight (LMW) peptides may have higher ACEI activity than high-molecular-weight (HMW) peptides. In a previous study [25], LMW chemical compounds were described that can reduce or completely inhibit the catalytic activity of their target enzyme(s). In this study, HMPH3 inhibited ACE, while HMPH10 relatively slightly inhibited ACE. Furthermore, as mentioned above, HMPH3 and HMPH10 have high contents of Glu, Asp, and Leu; it might contribute to ACEI activity resulting from their characteristic having negative electric charge and hydrophobic amino acid. Particularly, amino acids such as Glu, which has a carboxylate anion at the C-terminal, have ACEI activity. This can be explained by the capability of peptides rich in glutamic acid to chelate zinc being an element of the ACE active center [26]. Therefore, this finding implies that lower-molecular-weight peptides with hydrophilic amino acids such as Asp and Glu and hydrophobic amino acid such as Leu may act as competitive inhibitors (which interrupt with the enzyme active position so that the substrate cannot bind) or noncompetitive inhibitors (which change the enzyme shape so that the substrate cannot bind). However, this study suggests the need for further study such as Lineweaver-Burk plot method being able to determine ACE inhibitor type (competitive inhibitors or noncompetitive inhibitors).

This study showed the antioxidant activities of peptides of HMPH. The results of several antioxidant assays indicated that HMPH had antioxidative activity regardless of its molecular weight. HMPH3 showed a metal ion chelating effect and nitrite scavenging activity in a dose-dependent manner in this study. Transition metals including Cu^2+^ and Fe^2+^ can catalyze the production of reactive oxygen species (ROS) such as hydroxyl radical (^•^OH) and the superoxide anion (O_2_^−^). Fe^2+^ causes ^•^OH production by the Fenton reaction, and this radical can initiate a lipid peroxidation chain reaction. Thus, the chelating reaction of metal ions can contribute to antioxidative activity. In this study, HMPH3 and HMPH10 both showed a dose-dependent iron chelating activity. This result is in agreement with a previous study [27], which reported that the antioxidative peptides isolated from the hydrolysate were acidic peptides including acidic amino acids in their sequences. Canabady-Rochelle et al. (2015) also presented that the sequences of peptides are linked to their metal chelating ability [28]. Thus, we assume that anions of the acidic amino acids (Glu and ASP) would interact with cations of Fe(II) and then inactivate them, preventing them from having prooxidant effects. Di Bernardini et al. (2011) also revealed an antioxidant activity of hydrolysates from muscle protein sources using* in vitro* assays (ABTS, DPPH, iron chelation [29], and nitrite scavenging assays). Moreover, hydrolysates from grass carp muscle under 3 to 10 kDa were shown to have antioxidant activities [30]. The acidic amino acids with negative charged such as Glu and ASP show free radical quenching activity due to the existence of excess electrons [31]. Chen et al. (2015) also reported on peptides that showed a high reducing power, scavenging of free radicals, and reduction of lipid peroxidation, whereas showing a low ferrous iron chelating activity [32]. In this study, the main amino acids of HMPH were negatively charged Glu and ASP; therefore, these amino acids could act as antioxidants due to having a free radical quenching activity.

In this study, the ion chelating and nitrite scavenging activities of HMPH3 were higher than those in HMPH10, whereas DPPH radical scavenging activity was higher in HMPH10. These results indicate that antioxidative activities could be influenced by the molecular weight of HMPH. In a previous study, Ajibola et al. (2011) showed that a LMW peptide derived from African yam bean seed protein had a higher DPPH scavenging activity than a HMW peptide from the same source [33]. They suggested that the higher DPPH radical scavenging activity of the LMW peptide may be due to its content of hydrophobic and aromatic amino acids, which facilitate interaction with free radicals to discontinue their activities [33]. Although the exact mechanisms underlying the differences in antioxidative activity among peptides with different amino acid sequences are still largely unknown, we assume that the chemical structure and amino acid composition of peptides may be the main elements that influence their antioxidative activities. Therefore, more studies to determine the optimum molecular weight of HMPH are needed to develop it as a potential antioxidant in the future.

In this study, RAW 264.7 cell viability was unaffected by peptides of HMPH at concentrations of 5–20 mg/mL. However, the cell viability decreased at concentrations above 40 mg/mL, regardless of the molecular weight of HMPH. Although the cytotoxic mechanism of HMPH at 40 mg/mL is unclear, De Mejia and De Lumen (2006) proposed a mechanism whereby the peptide selectively kills transformed cells through binding to deacetylated core histones to disrupt the dynamics of histone acetylation-deacetylation [34]. Moreover, Nurdiani et al. (2017) investigated different fractions of peptides derived from flathead fish and found that the LMW fraction (<3 kDa) had the highest cytotoxicity among fractions [35]. When cytotoxicity exceeds appropriate levels, selectivity between eukaryotic and prokaryotic cells is lost [36]. In addition, the hydrophobicity of peptides could strongly influence their cytotoxicity. Liu et al. (2016) reported that peptides containing hydrophobic amino acids arranged in amphipathic *α*-helices or *β*-structures displayed a potent cell-killing effect [37]. Most cationic antimicrobial peptides result in cell death through membranolytic mechanisms [38], in which the mitochondrial membrane or plasma is selectively disrupted [39]. Another study documented that membranolytic anticancer peptides, which have a content of 40–60% hydrophobic amino acids and contain 10–30 amino acids, exert their cytotoxicity by receptor-independent membrane disruption. Gabernet et al. (2016) summarized three membrane disruption models for cell cytotoxicity: first, the barrel-stave pore (membranolytic peptides form a pore in a perpendicular orientation to the membrane surface by directly interacting with the phospholipid acyl chains); second, the toroidal pore (which contacts the phospholipid head groups); third, carpet and detergent-like mechanisms (which cause membrane blebbing) [38].

As mentioned above, hydrophobic amino acids are known to be related to cytotoxicity. This study showed that the cell viability of RAW 264.7 cells decreased at dose of peptides of HMPH above 40 mg/mL. This result may stem from the concentration of hydrophobic amino acids reaching a cytotoxic level when the peptides of HMPH concentration were increased. Therefore, the authors assume that the hydrophobicity or polarity of amino acids in HMPH could affect cell viability. The present study revealed that different amino acid compositions and different molecular weights of HMPH differed in their ACEI and antioxidative activities; moreover, a high concentration of peptides of HMPH showed cytotoxicity against murine macrophages. Therefore, the molecular weight, amino acid composition, and concentration should be optimized to further improve the ACEI and antioxidative activities of HMPH.

## 5. Conclusions

This study determined that peptides of HMPH have an ACE inhibitory effect and an antioxidant activity. HMPH was obtained by hydrolysis using a commercial enzyme (Alkaline-AK), and its ACE inhibitory effect varied between peptides of HMPH with different molecular weights (<3 and 10 kDa), with the low-molecular-weight variant showing higher activity at above 20 mg/mL. Low-molecular-weight HMPH (<3 kDa) also showed higher iron chelating and nitrite scavenging activities, while high-molecular weight HMPH (<10 kDa) had a higher DPPH radical scavenging activity. The peptides of HMPH did not affect RAW 264.7 cell viability at concentrations of 5–20 mg/mL. Based on the results of this study, the authors assume that the ACE inhibitory and antioxidant activities of peptides of HMPH are influenced by hydrophilic amino acids with a negative charge. Various bioactive peptides have been produced using enzymes such as alcalase, neutrase, proteinase K, or trypsin. These enzymes are expensive due to the requirement for a high purity of each enzyme, and the enzyme Alkaline-AK used in this study is approximately 270,000 times cheaper than the other reported enzymes. Therefore, Alkaline-AK may be economically viable for use in the production of bioactive peptides, and HMPH represents a potential ACE inhibitor and antioxidant.

## Figures and Tables

**Table 1:** Proximate compositions of myofibrillar protein and HMPH.

	Crude moisture	Crude ash	Crude protein	Crude fat
	Content (%)			
Myofibrillar protein	74.41 ± 1.58	0.18 ± 0.02	26.99 ± 2.45	2.12 ± 0.84
HMPH^(1)^	3.39 ± 1.64	0.78 ± 0.02	73.62 ± 2.44	19.28 ± 1.18

^(1)^HMPH: hydrolyzed myofibrillar protein from Hanwoo cattle.
